# Joint Rare Variant Association Test of the Average and Individual Effects for Sequencing Studies

**DOI:** 10.1371/journal.pone.0032485

**Published:** 2012-03-16

**Authors:** Yuanjia Wang, Yin-Hsiu Chen, Qiong Yang

**Affiliations:** 1 Department of Biostatistics, Mailman School of Public Health, Columbia University, New York, New York, United States of America; 2 Department of Statistics, Columbia University, New York, New York, United States of America; 3 Department of Biostatistics, Boston University, Boston, Massachussetts, United States of America; Memorial Sloan Kettering Cancer Center, United States of America

## Abstract

For many complex traits, single nucleotide polymorphisms (SNPs) identified from genome-wide association studies (GWAS) only explain a small percentage of heritability. Next generation sequencing technology makes it possible to explore unexplained heritability by identifying rare variants (RVs). Existing tests designed for RVs look for optimal strategies to combine information across multiple variants. Many of the tests have good power when the true underlying associations are either in the same direction or in opposite directions. We propose three tests for examining the association between a phenotype and RVs, where two of them jointly consider the common association across RVs and the individual deviations from the common effect. On one hand, similar to some of the best existing methods, the individual deviations are modeled as random effects to borrow information across multiple RVs. On the other hand, unlike the existing methods which pool individual effects towards zero, we pool them towards a possibly non-zero common effect by adding a pooled variant into the model. The common effect and the individual effects are jointly tested. We show through extensive simulations that at least one of the three tests proposed here is the most powerful or very close to being the most powerful in various settings of true models. This is appealing in practice because the direction and size of the true effects of the associated RVs are unknown. Researchers can apply the developed tests to improve power under a wide range of true models.

## Introduction

Genome-wide association studies (GWAS) utilizing common single nucleotide polymorphisms (SNPs) have been successful in identifying genetic variants associated with various diseases and complex traits [1]. However, for many complex traits, heritability explained by identified SNPs is low [2]. It has been hypothesized that some of the heritability may be explained by previously un-identified rare variants [3]–[5]. With the advances of next-generation sequencing technology, data from targeted or whole genome sequencing is being produced where many of sequencing variants are rare, e.g., minor allele frequency (MAF) less than 1%.

Since rare variants (RVs) have extremely low frequencies, traditional single-variant based analysis for GWAS is under-powered for detecting rare variants. In recent literature, various approaches specifically designed for rare variants are proposed and compared [6]–[11]. Most approaches involve combining information across rare variants. For example, the Sum-Test proposed by Li and Leal [6] and other similar approaches collapse multiple rare variants into a single “super” variant [7]–[8] through a weighted average, and test the association between the “super variant” and the trait. The motivation of these tests is to minimize the cost of the degrees of freedom of the association test. They have the best power when the effects of all genetic variants are in the same direction. The power diminishes if the true associations vary across variants in opposite directions.

Pan [12] proposed a summed score test (SSU) motivated from combining squared score statistics across multiple variants. It was found to be equivalent to testing a variance component in a random effects model [13] with a binary outcome when permutation is used to compute its 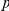-value [12]. This model assumes that the association parameters are random effects centered around zero. Thus the average association across variants is zero. Wu, et al. [10], [14] proposed kernel based tests (SKAT) that exploit information across variants using kernel machines. The connection between SSU and SKAT also lies in testing a variance component in a random effects model. Lin and Tang [15] recently proposed an extension of the Sum-Test, named EREC, where each variant is weighted by its estimated effect size plus a constant. As noted by the authors, a very large sample size is required to achieve the asymptotic normality and optimality of the test statistics. In addition, the choice of the constant is rather arbitrary. Basu and Pan [11] conducted extensive simulations to compare eight RV tests including the Sum-Test, SSU, and SKAT with binary traits under numerous settings. They concluded that SSU and SKAT are among the best across many settings. None of the existing RV tests suitable for associations in opposite directions considers testing both the common association and the individual deviations from the common association.

Here, we first propose a joint likelihood ratio test (LRT-Joint) and a joint score test (Score-Joint) for a common association and individual associations of the rare variants with a continuous or binary trait. The test is motivated by taking advantage of both the Sum-Test and the SSU (or SKAT). Essentially, we add a pooled super variant into the model. On one hand, like the SSU or SKAT, the individual associations are modeled as random effects to borrow information across multiple RVs. On the other hand, unlike these two existing methods which pool the individual effects to zero, we pool them towards a possibly non-zero common effect (or average effect) as in the Sum-Test. Secondly, we examine a restricted likelihood ratio test (RLRT) of individual associations in a random effects model. We compare the proposed tests with the Sum-Test, SSU, and SKAT by extensive simulations and show that in many cases, at least one of the proposed tests outperform existing ones that ignore either the common association or the individual effects. All proposed tests are fast to compute and easy to implement with standard statistical softwares such as R or SAS.

## Methods

### Continuous trait

We start by introducing methods for the continuous trait. Let 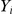 denote the outcome, and let 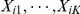 denote the number of minor alleles for each RV. Assume subjects' outcomes are independent and assume an additive genetic model. There are two extreme modeling strategies, as noted in Pan [12]. One strategy is to jointly model all variants by a linear regression,
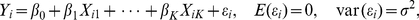
(1)


and the hypothesis of no association in the above model is
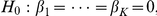



which involves a test of 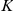 degrees of freedom. Such a test may have very low power when 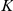 is large and some of the RVs included have no association with the trait. To improve power, a possibly misspecified working model is to assume that all the RVs have the same magnitude of association with the outcome, that is, 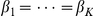. Let 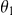 denote this common association effect. Then the model is
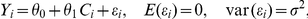



where 
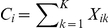
 is considered as a “super variant” [7]–[8]. Under this working model, no association is tested by
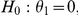



which involves only one degree of freedom. The resulting test is referred to as the Sum-Test [6]. It is expected that the Sum-Test is most powerful when the working model assumption of 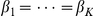 is correct. However, when the assumption is incorrect, especially when the associations are in opposite directions, the Sum-Test suffers from significant power loss.

Pan [12] proposed the SSU test as
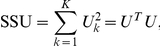



where 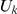 is the score statistic obtained from a marginal model of 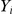 on 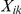, and 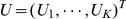. The SSU was shown to improve the power compared to the Sum-Test when the associations are in opposite directions [11]. Furthermore, Pan [12] showed that SSU is related to Goeman, et al. [13], which is designed for testing a high dimensional alternative. Assuming 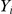
's are centered, then the Goeman's test is the score statistic derived from a random effects model
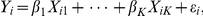


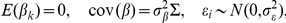



where 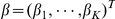 are random effects independent of 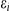, and 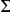 is a semi-positive-definite covariance matrix. Note that no distributional assumption is placed on 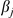. The test statistic is
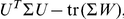



where 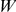 is the observed Fisher's information matrix of 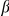 under 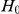. In such a random effects model, the null hypothesis of no association with any of the 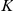 variants is
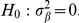



The test pertains to a single parameter instead of 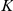 parameters in (1). If computing the critical value of the SSU using permutation and choosing 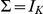, then the SSU is equivalent to the Goeman's test [12].

Here we propose several tests that combine the advantages of the Sum-Test and SSU (or the Goeman's test). Note that the Goeman's test is derived under the assumption that the means of 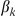 are all zero, therefore all 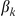 are pooled towards zero when 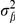 is small. However, unless the effect of all rare variants are in opposite directions with the same strength and therefore they cancel out, the average effect will not be zero. Thus, a model restricting the average effect to be zero may have lower power than a more flexible model.

We propose a score test based on the model
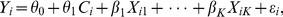
(2)

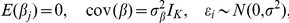



where 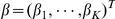. Note that in this model, 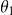 represents the average effect across all RVs, and 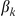 represents the deviation of the 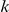
th RV from the common effect. Essentially, we add the super variant 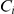 as a fixed effect into the model. No distributional assumption about 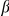 is placed. The null hypothesis of no association between the phenotype and any of the RVs is
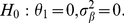
(3)


Note that testing only 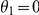 in model (2) is not equivalent to the Sum-Test due to the presence of 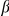 if the true 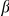 is not all zero.

In the Supporting Information S1, we show that the score for testing 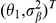 is
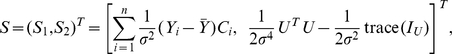



where 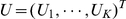, 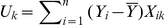, and 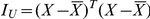. The score test statistic for hypothesis (3) in model (2) is (Score-Joint)
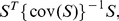
(4)


and we show in the Supporting Information S1

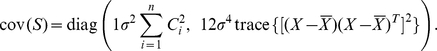



Under the null hypothesis, 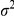 is estimated by the sample variance of 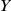. Note that since 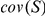 is a diagonal matrix, the score statistic is simply
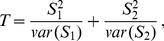
(5)


which standardizes each component of 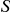 by its variance and assigns equal weight to both components. Since the variance component in hypothesis (3) is on the boundary of the parameter space under the null, the usual chi-square approximation does not apply. We use permutation to compute the 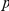-value of the test. Note that the score statistic only involves matrix manipulations and is fast to compute.

Next, we describe two likelihood based tests where distributional assumption is placed on 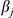
's. The first one is a likelihood ratio test (LRT) for testing the null hypothesis (3) in the model (2) with an additional assumption that 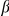 has a multivariate normal distribution, which we refer as LRT-Joint. Crainiceanu and Ruppert [16] found that the LRT involving a variance component does not follow a standard chi-square mixture distribution and using the usual 50∶50 chi-square mixture can be conservative. We use permutation to compute the 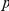-value of the LRT-Joint. The second likelihood based test is a restricted likelihood ratio test (RLRT) of a variance component, that is, to test
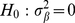
(6)


in a model not accounting for the common association, i.e.,
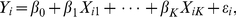
(7)

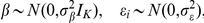



where 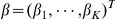. This can be regarded as the RLRT version of the SSU and the Goeman's test. Again, due to a non-standard null distribution, we use permutation to compute the null distribution of the RLRT.

Among three proposed tests, the Score-Joint and LRT-Joint are two variations of the likelihood-based joint tests of the common and individual effects. They are two tests of the same hypothesis (3) under slightly different model. The RLRT only tests the individual effects, i.e., hypothesis (6), in model (7) through the restricted maximum likelihood. Similar to the Goeman's test, the Score-Joint here does not assume multivariate normality while the LRT-Joint and RLRT do.

### Binary traits

Many genetic studies involve binary outcomes such as the presence or absence of a disease. A popular model used to analyze binary data is the logistic model. We propose a similar joint score test for the binary trait based on the logistic regression. The model for binary data corresponding to (2) is
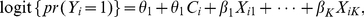
(8)

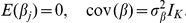



We show in the Supporting Information S1 that the score vector for the parameters 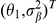 is
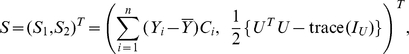



where 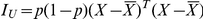 and 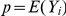. Under the null hypothesis, 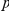 is estimated as 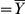 and 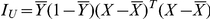. We compute the covariance matrix of the score vector in the online Supporting Information S1. The score statistic 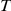 is defined similarly as the continuous case in (4).

It is straightforward to carry out the LRT and RLRT for the binary outcome under the extra assumption of the multivariate normality of the random effects (for example, using SAS procedure GLIMMIX). However, since both of these tests involve a variance component, similar to the continuous outcome case, their null distributions are non-standard. Therefore, we also use permutation to compute the 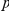-value of the LRT-Joint and RLRT.

## Results

### Simulation settings and methods

We designed our simulation studies following procedures similar to that of Basu and Pan [11]. We generated samples with 1000 subjects. We simulated 10 RVs associated with the disease and 0, 5, 10, 20 or 30 neutral variants (NVs) that do not associate with the disease. To simulate correlated RVs, we generated a 10-dimensional latent continuous vector from a multivariate normal distribution with AR(1) correlation structure. The autocorrelation 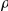 was set as 0, 0.5 or 0.8 to represent no correlation, moderate correlation and strong correlation. We created two independent haplotypes dichotomized from the latent multivariate normal random variable. The threshold for dichotomization was chosen such that the haplotypes will have the MAFs randomly drawn from a uniform distribution with support between 0.001 and 0.01. The two haplotypes were then combined to create genotypes.

The continuous outcomes were simulated from model (1) with standard normal random errors. The binary outcomes were generated from a logistic model 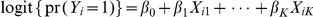. We considered five different settings for the coefficients of genetic associations. Each setting corresponds to a different combination of 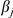 ranging from one extreme of taking the same value to the other extreme of taking exactly opposite values. For the continuous case, the average effect across ten disease associated SNPs (i.e., 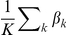) ranges from zero to 0.4 (a small effect size). For the binary case, the average odds ratio across ten SNPs ranges from 1.0 to 2.0. For each set of 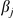, we simulated three different autocorrelations (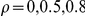) and five different numbers of NVs (0, 5, 10, 20, and 30).

**Table 1:** Type I error rate for all tests: continuous trait, 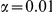.

No. of NV	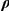	LRT-joint	Score-joint	Sum Test	LRT-single	SumSqB	SKAT
0	0	0.012	0.011	0.013	0.010	0.009	0.008
0	0.5	0.012	0.012	0.014	0.012	0.008	0.014
0	0.8	0.012	0.011	0.010	0.011	0.011	0.012
5	0	0.012	0.012	0.014	0.012	0.008	0.012
5	0.5	0.013	0.010	0.014	0.006	0.006	0.014
5	0.8	0.008	0.009	0.008	0.010	0.006	0.010
10	0	0.005	0.010	0.006	0.010	0.014	0.012
10	0.5	0.008	0.007	0.006	0.012	0.010	0.008
10	0.8	0.013	0.007	0.014	0.010	0.008	0.016
20	0	0.008	0.015	0.006	0.008	0.010	0.012
20	0.5	0.006	0.010	0.009	0.013	0.013	0.016
20	0.8	0.008	0.008	0.009	0.008	0.007	0.014
30	0	0.010	0.010	0.007	0.010	0.010	0.010
30	0.5	0.008	0.008	0.010	0.010	0.009	0.006
30	0.8	0.007	0.009	0.004	0.014	0.012	0.014

**Table 2:** Type I error rate for all tests: continuous trait, 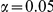.

No. of NV	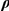	LRT-joint	Score-joint	Sum Test	LRT-single	SumSqB	SKAT
0	0	0.048	0.046	0.050	0.050	0.056	0.052
0	0.5	0.043	0.042	0.044	0.058	0.052	0.048
0	0.8	0.040	0.048	0.043	0.058	0.046	0.066
5	0	0.042	0.042	0.042	0.052	0.056	0.062
5	0.5	0.054	0.046	0.056	0.056	0.060	0.044
5	0.8	0.056	0.046	0.058	0.040	0.042	0.056
10	0	0.048	0.056	0.040	0.058	0.050	0.042
10	0.5	0.050	0.058	0.060	0.040	0.034	0.054
10	0.8	0.044	0.046	0.054	0.046	0.048	0.054
20	0	0.056	0.052	0.056	0.044	0.042	0.038
20	0.5	0.040	0.058	0.048	0.044	0.048	0.040
20	0.8	0.042	0.044	0.056	0.048	0.040	0.064
30	0	0.046	0.048	0.040	0.056	0.056	0.072
30	0.5	0.048	0.054	0.051	0.046	0.050	0.048
30	0.8	0.052	0.050	0.047	0.048	0.044	0.050

To compute the null distribution of a test statistic by permutation, we randomly permuted the outcome among all subjects 2000 times and determined the critical value as the desired percentile from the empirical distribution of the permuted test statistic. We then computed the proportion of the test statistics simulated under the null (or the alternative) greater than or equal to the threshold as the empirical type I error rate (or power). We computed the 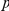-value as the proportion of the permuted test statistics greater than or equal to the simulated observed test statistic.

**Table 3:** Type I error rate for all tests: binary trait, 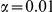.

No. of NV	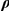	LRT-joint	Score-joint	Sum Test	LRT-single	SumSqB	SKAT
0	0	0.006	0.008	0.006	0.008	0.009	0.010
0	0.5	0.008	0.008	0.005	0.010	0.007	0.010
0	0.8	0.006	0.013	0.006	0.006	0.011	0.008
5	0	0.008	0.006	0.011	0.009	0.006	0.007
5	0.5	0.012	0.010	0.010	0.008	0.007	0.010
5	0.8	0.010	0.012	0.007	0.010	0.008	0.018
10	0	0.010	0.013	0.009	0.008	0.013	0.008
10	0.5	0.008	0.009	0.008	0.010	0.007	0.006
10	0.8	0.006	0.008	0.005	0.010	0.007	0.008
20	0	0.006	0.013	0.006	0.008	0.009	0.014
20	0.5	0.010	0.006	0.008	0.012	0.011	0.014
20	0.8	0.014	0.010	0.006	0.010	0.008	0.012
30	0	0.008	0.007	0.006	0.012	0.005	0.010
30	0.5	0.016	0.012	0.006	0.014	0.007	0.006
30	0.8	0.008	0.010	0.011	0.006	0.010	0.006

We compared six tests by simulations. The first is the LRT-Joint of both the common and individual RV effects. The second is the Score-Joint in (4).The third is the RLRT to test the individual effects through the hypothesis (6) of a variance component. The fourth test is the Sum-Test which is most suitable for testing the common association effect. The fifth test is the SSU for individual effects proposed in Pan, [12]. The last test is the SKAT with weighted linear kernel (Beta distribution as weights with default parameters) and Davies [17] (implemented in SKAT) to compute the 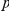-value. For each setting, we ran 1000 permutations for the proposed tests and 500 replications to examine the empirical type I error or power.

**Table 4:** Type I error rate for all tests: binary trait, 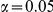.

No. of NV	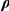	LRT-joint	Score-joint	Sum Test	LRT-single	SumSqB	SKAT
0	0	0.052	0.044	0.060	0.048	0.044	0.050
0	0.5	0.056	0.058	0.048	0.060	0.044	0.056
0	0.8	0.044	0.046	0.050	0.034	0.032	0.048
5	0	0.050	0.048	0.052	0.052	0.044	0.062
5	0.5	0.060	0.046	0.054	0.062	0.052	0.048
5	0.8	0.048	0.052	0.042	0.058	0.042	0.050
10	0	0.046	0.040	0.048	0.044	0.038	0.056
10	0.5	0.050	0.050	0.042	0.050	0.034	0.056
10	0.8	0.058	0.046	0.050	0.060	0.038	0.052
20	0	0.046	0.052	0.066	0.036	0.038	0.044
20	0.5	0.056	0.052	0.032	0.050	0.060	0.050
20	0.8	0.046	0.060	0.036	0.046	0.032	0.042
30	0	0.058	0.060	0.058	0.062	0.048	0.042
30	0.5	0.046	0.044	0.048	0.042	0.044	0.056
30	0.8	0.038	0.042	0.036	0.046	0.040	0.046

**Figure 1:**
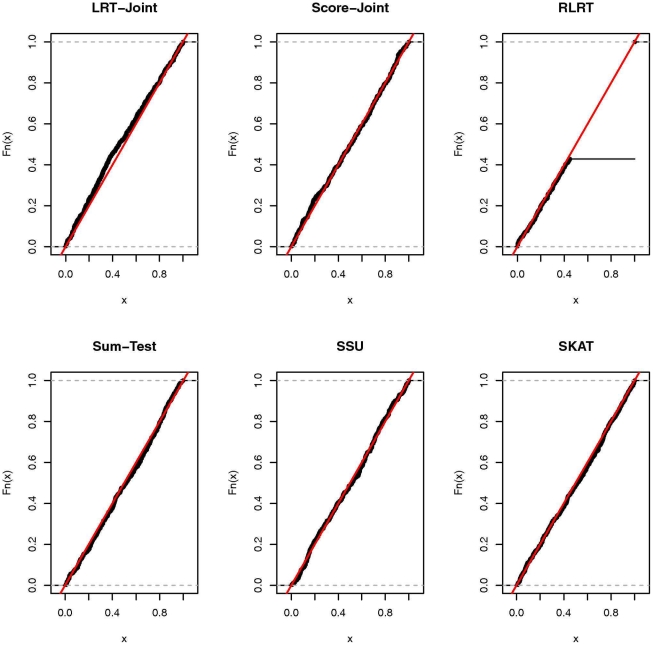
Empirical cumulative distribution function (ECDF) of the 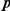-values under the null for continuous trait.

### Simulation results

**Figure 2:**
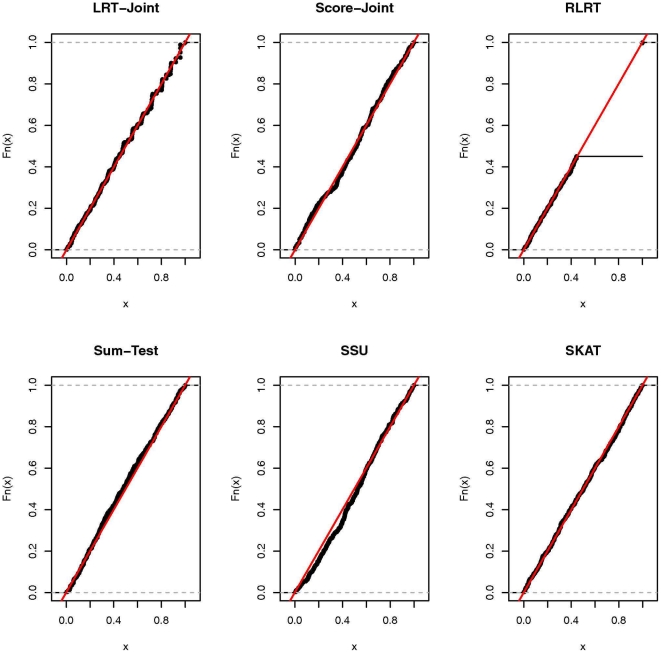
Empirical cumulative distribution function (ECDF) of the 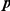-values under the null for binary trait.

First, we assessed the type I error rate of all the tests. In Tables 1 and 2, we present the empirical type I error rates for the continuous trait at 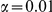 and 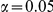, respectively. In Tables 3 and 4, we present the type I error rates for the binary trait. These four tables show that all tests adhere to the nominal level for both types of traits at each significance level. To examine the distribution of the 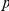-values under the null, we present the empirical cumulative distribution function (ECDF) of the 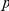-values for all six tests with the continuous and binary trait in Figures 1 and 2. Except for the RLRT, the expected distribution of the other five tests is a uniform distribution between zero and one. For the RLRT, the null distribution of the test statistic is non-standard due to that the parameter being tested (i.e., 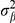) is on the boundary of the parameter space under the null and the non-independence of the response variable under the alternative model [16]. The null distribution of the RLRT test statistics is a mixture of 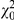 (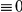) and 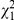, i.e., 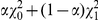. Since 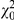 is a degenerated distribution that is exactly zero, a proportion of the RLRT test statistics are zero (RLRT has a point mass at zero). Therefore the distribution of the 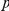-values is also non-standard: it has a point mass (which equals to 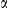) at one and has a uniform distribution between zero and 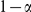. The null distribution of LRT-Joint test statistics is also a mixture of 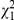 and 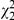. However, since 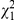 is non-degenerated, the distribution of the 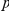-values does not have a point mass at one.

**Figure 3:**
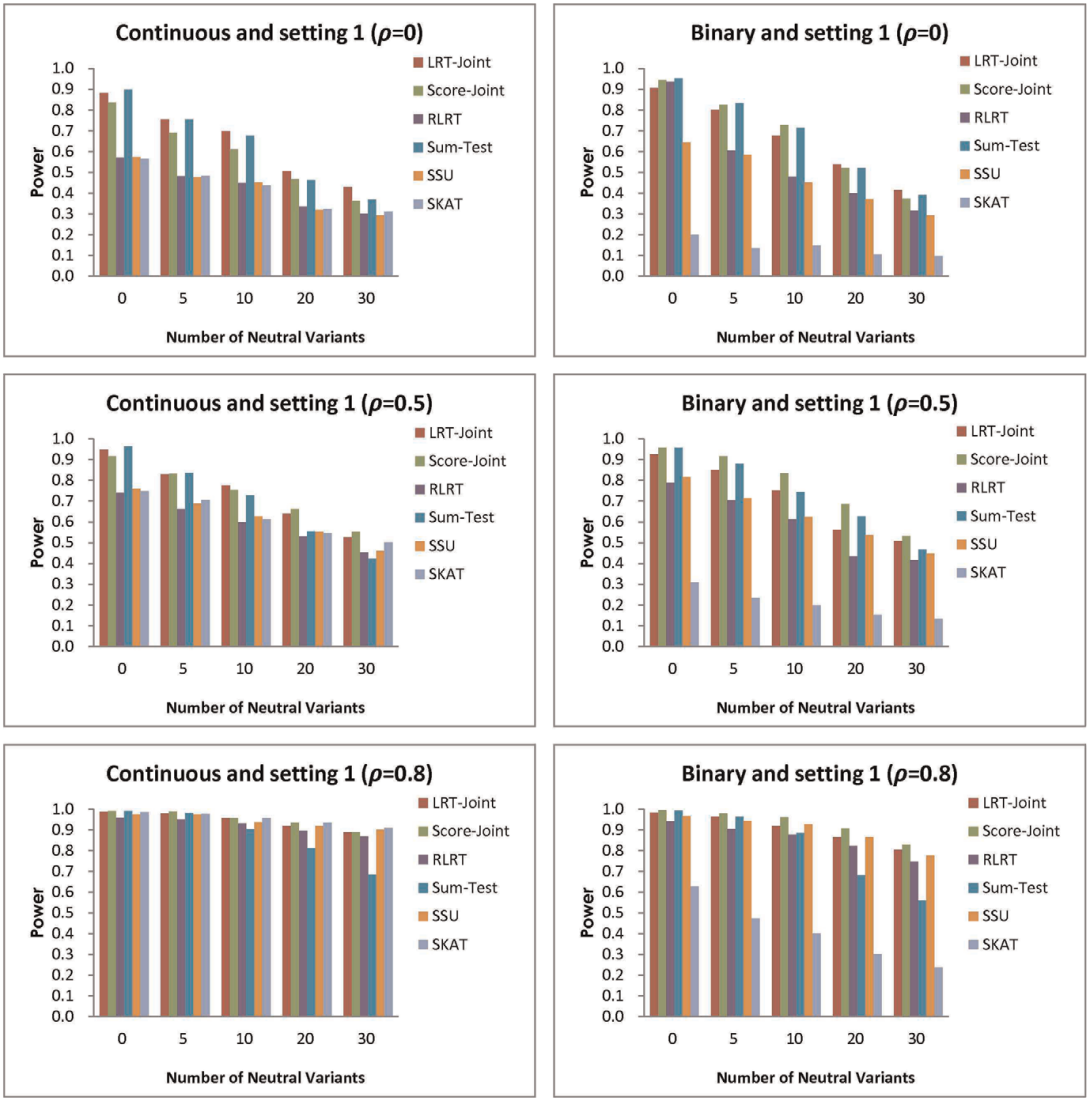
Simulation setting 1: 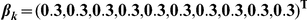 (continuous trait), and OR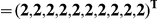 (binary trait).

To compare power with 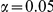, we designed five settings with different magnitude of the average common effect and individual SNP effects. In the first setting, the coefficients for all the SNPs are the same: 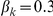 for the continuous trait and OR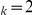 for the binary trait. Thus, this setting favors the tests involving the common effect. We summarize the results in Figure 3. When there are no neutral variants, no correlation (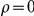) and continuous traits, the Sum-Test has the best power and is closely followed by the LRT-Joint and Score-Joint (Figure 3). The RLRT, SSU and SKAT do not test the common association and have much less power in this case (about 30% power loss). Still, with no NV but with an increasing correlation, the LRT-Joint, Score-Joint and Sum-Test continue to have comparable high power, while the difference between them and the RLRT, SSU and SKAT gets smaller. When there are NVs included, the power of the Sum-Test can be much less than the LRT-Joint or Score-Joint, especially when 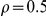 and 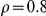 and NV = 20 or NV = 30. This is because since the NVs have zero association, even though the disease associated RVs have the same association, the average effect across all RVs included in the test statistic is decreased.

For the binary trait, the power comparison between the first five tests follow a similar trend. The SKAT has much less power than the other tests in this setting. The Score-Joint has the best power among all tests in most scenarios. The RLRT and SSU are inferior to the LRT-Joint and Score-Joint. It is worth to note that in this setting, for both the continuous and binary traits, stronger correlation leads to increased power for all tests. This is because the associations are in the same direction for all RVs and stronger correlation among RVs happens to strengthen similarity between the effects of RVs.

**Figure 4:**
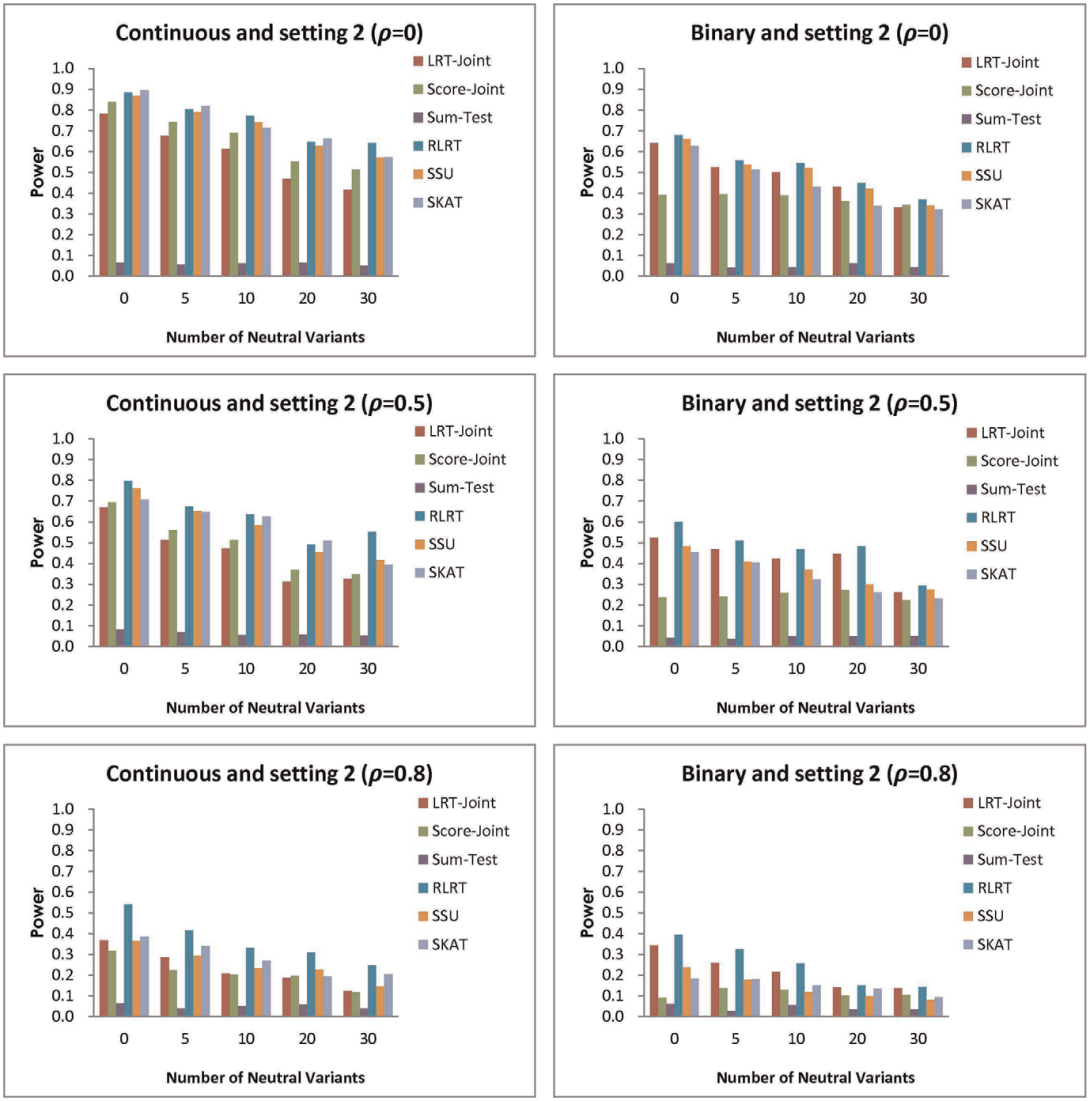
Simulation setting 2: 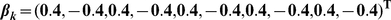 (continuous trait) and OR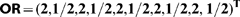 (binary trait).

In the second setting, the association parameters have opposite signs with the same magnitude and the average effect across all RVs is zero. Since this setting is least favorable for tests involving common effect, as expected the Sum-Test has little power. For the continuous trait, the RLRT, SSU and SKAT have similar power across different scenarios of correlation and number of NVs (Figure 4). The Score-Joint performs better than LRT-Joint in many cases, but has less power than the RLRT, SSU or SKAT. This reflects a loss in power for testing the extra common association parameter of the joint tests when the true average association is zero. For the binary trait, the RLRT is the most powerful and is closely followed by the LRT-Joint in most cases. Note that in this case, greater correlation resulted in lower power for all tests. This is because the true associations are not in the same direction, while more correlation encourages similar fit among parameters in the model.

**Figure 5:**
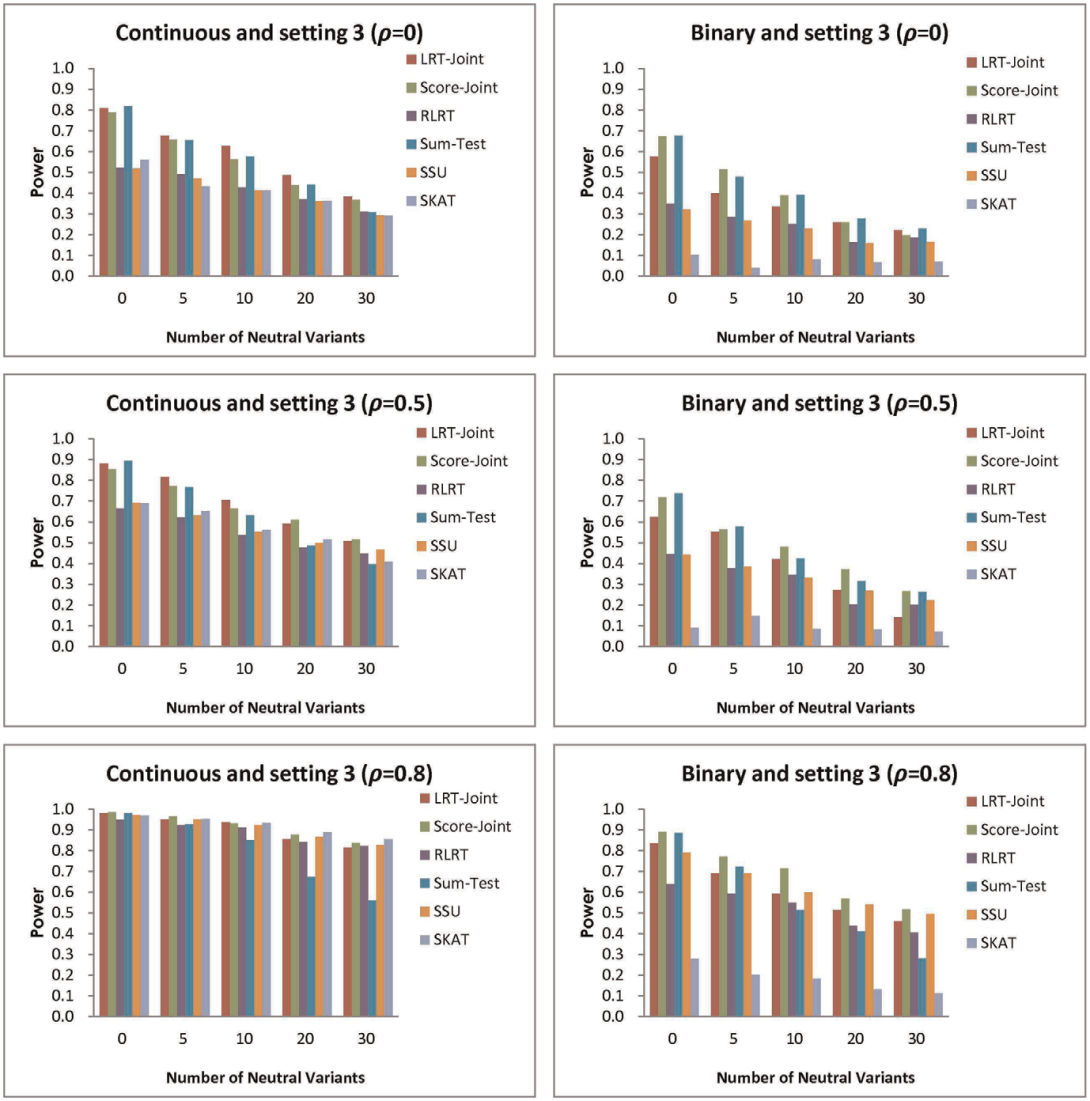
Simulation setting 3: 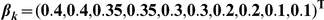 (continuous trait); OR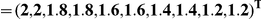 (binary trait).

In the third setting, all the associations are in the same direction, but with varying magnitude. Similar to the first setting, the two joint tests involving common association effect outperform the SSU, RLRT, and SKAT, which only test for individual effects (Figure 5). The difference in the power is smaller when there are stronger correlation or more NVs. The Sum-Test performs worse than the two joint tests when there is a large number of NVs. For the continuous trait, the power of the LRT-Joint and Score-Joint are close to the best or the best across multiple settings. For the binary trait, in a majority of the scenarios, the Score-Joint outperforms all other tests with the exception of two cases where the Sum-Test is slightly more powerful.

**Figure 6:**
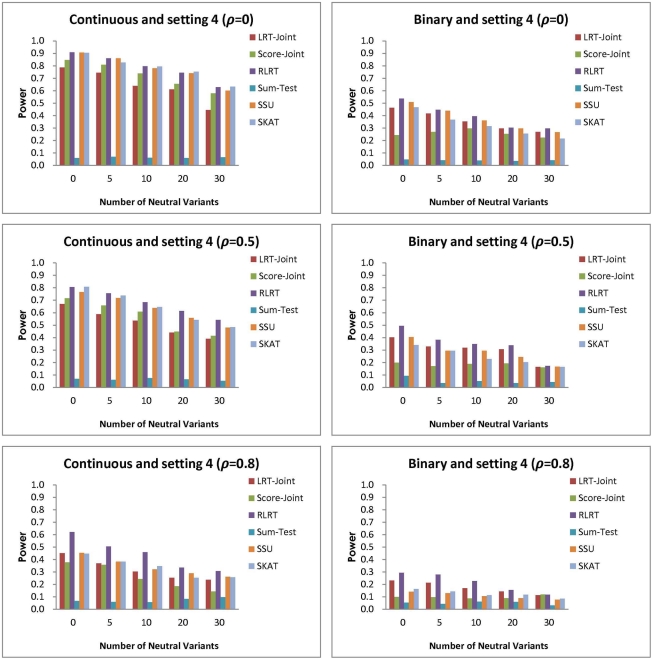
Simulation setting 4: 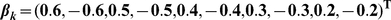 (continuous trait); OR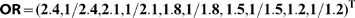 (binary trait).

In the fourth setting, the associations are in opposite directions with varying magnitudes, and the average effect across all RVs is again zero. As expected, the Sum-Test has no power in all the scenarios here (Figure 6). The RLRT is the most powerful across a majority of the scenarios in this setting. For the continuous trait, the second best test is usually the SSU or SKAT. With no correlation or moderate correlation, the difference between the RLRT and the second best test is modest. However, with strong correlation, the difference between the RLRT and SSU can be as large as 17%. For the binary trait, the second most powerful test is the LRT-Joint.

**Figure 7:**
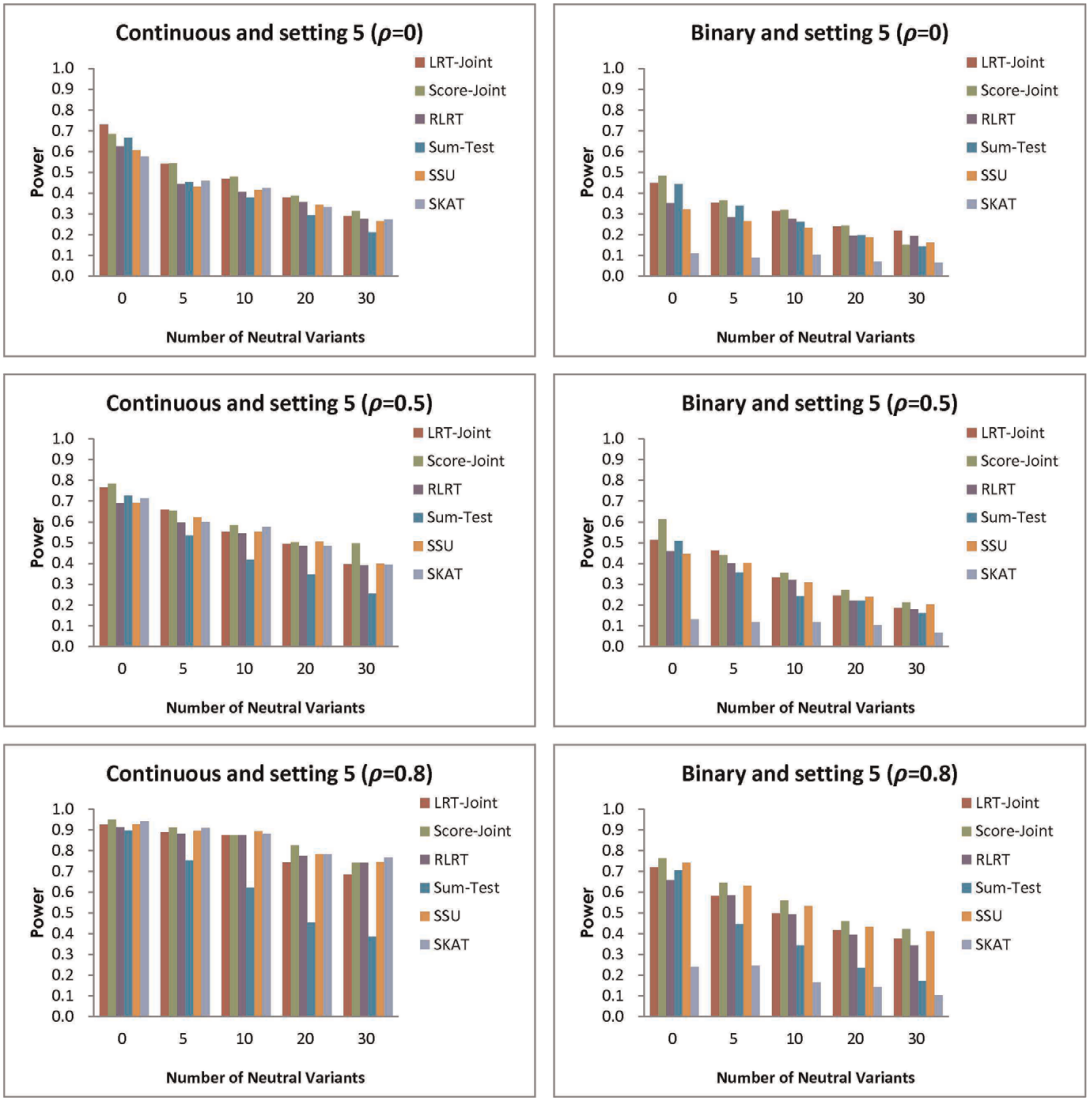
Simulation setting 5: 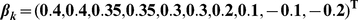 (continuous trait); OR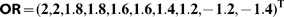 (binary trait).

The fifth setting is a mixture of the above settings where the majority of the association parameters is in the same direction and only two of them are in the opposite direction. Again, when NVs are present, the Sum-Test is clearly inferior to the other tests (Figure 7). In most scenarios, the Score-Joint is the most powerful test for both traits. For the continuous trait with strong correlation, the Score-Joint, RLRT and SSU all have similar power and outperform the LRT-Joint. With the binary trait and strong correlation, the Score-Joint and SSU have similar power and outperform the other tests. The SKAT has low power in this setting.

## Discussion

In this work, we propose three new tests (Score-Joint, LRT-Joint, and RLRT) for detecting disease association with RVs. The two joint tests examine both a common association across RVs and individual deviations from the common effect. The RLRT we propose only examines the individual effects. When the true underlying disease model includes RVs with the same association effect, the two joint tests outperform RLRT, SKAT, or SSU, especially with no correlation or moderate correlation among RVs. This reflects the benefit of considering the average association in the joint tests and the power loss without accounting for such effects in the RLRT, SKAT and SSU. Also note that in cases where there are no individual deviations among disease associated RVs, the loss in power for testing an extra variance component in the joint tests comparing to the Sum-Test is minimal. In addition, the Sum-Test is only more powerful when there are no NVs. Thus, the joint tests are preferred in cases where there are NVs.

When the true underlying disease model includes RVs with associations in opposite directions but the same strength, the average effect is zero but the individual effects are not zero. The RLRT is the most powerful test in most scenarios. Note that even though SSU, SKAT are the score tests and RLRT is the restricted likelihood ratio test of the same hypothesis on the individual effects, they do not necessarily have the same power, especially for the binary trait. One difference between the models used to derive the RLRT and the SSU (or equivalently the Goeman's test) is that the RLRT assumes that the random effects follow a multivariate normal distribution, while the Goeman's test assumes the mean and covariance structure of the random effects but not the distribution. For the binary trait, the SSU or SKAT can be much less powerful than the RLRT, even though the multivariate normal random effects working assumption is not necessarily true.

When the strengths of the disease associated RVs have varying magnitude in opposite signs, there is no average effect but there are individual effects. In this case, we continue to see the RLRT as the leading candidate in terms of power. The difference between the RLRT and SSU is larger than the previous setting with RVs in opposite directions but with the same strength, especially when the correlation is strong. When the majority or all of the RVs in the true model are in the same direction and with varying strengths, both the average effect and individual effects are non-zero. In these cases, the Score-Joint is often the most powerful test.

In practice, the effect sizes of RVs are unknown, therefore it is difficult to choose a single most powerful method out of a large number of available tests. At least one of the three tests we propose here is always the most powerful or very close to being the most powerful test in different settings. This is appealing in practice because researchers can apply the three tests to achieve high power for a wide range of underlying models. All tests proposed here are easy to implement (codes available at www.columbia.edu/ 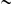
yw2016). For the binary trait, computing the LRT and RLRT may take slightly longer due to the need to fit a mixed effects model with generalized outcomes. Score tests do not require fitting a model under the alternative and therefore are slightly faster to fit. For example, with the binary trait, it takes about 8.3 seconds to perform LRT-Joint, 10.4 seconds for RLRT, and 1.4 second for Score-Joint (each test with 2000 permutations).

Our joint score test (5) assigns equal weight to each component of the score vector. It is conceivable that an adaptive version with carefully chosen weights may further improve power. However, if the weights also depend on outcomes, one may need to split samples to compute the weights and score statistics separately to correctly control the type I error rate. Since only a portion of the sample will be used to compute the score vector, there is a cost of using adaptive weights depending on the outcomes. Further research along this line is needed.

In summary, we proposed three new tests for rare variants that are among the most powerful tests, compared to several popular existing methods, across multiple scenarios. Our study reveals that it is worthwhile to jointly test the average association, as well as the individual deviations when there is a non-null average effect and there are NVs. This holds even when the individual deviations are absent. The Sum-Test solely considers average effect and does not perform well when there are NVs. When the average effect is zero, the RLRT appears to be the best test which can be more powerful than the SSU or SKAT.

## Supporting Information

Supporting Information S1 **Derivations of score statistic and its covariance matrix are in the online Supporting Information S1.**
(PDF)
