# Correction: Joint Rare Variant Association Test of the Average and Individual Effects for Sequencing Studies

**DOI:** 10.1371/annotation/9a117865-2fb1-4356-a0ec-fda016d471a1

**Published:** 2012-06-26

**Authors:** Yuanjia Wang, Yin-Hsiu Chen, Qiong Yang

There was an error in a grant number in the funding statement. R01NS073670-01 should be R01 NS073671-01. 

