# Supplementary material for: Joint Rare Variant Association Test of the Average and Individual Effects for Sequencing Studies
Source: PLoS One. 2012 Mar 16;7(3):e32485. doi: 10.1371/journal.pone.0032485 (PMC3309869; doi:10.1371/journal.pone.0032485)
Supplement: Supporting Information S1 — Derivations of score statistic and its covariance matrix are in the online Supporting Information S1. (PDF) [file pone.0032485.s001.pdf]

Online Appendix for "Joint rare variant association test of the average and individual effects for sequencing studies" by Wang, Chen and Yang

S1. Derivation of score statistic and its covariance matrix

First, we derive the joint score test for the continuous outcome. Taking the partial derivatives of the likelihood for model (2) with respective to θ_1 and σ_β^2, and using the results in Lemma 3 of Goeman et al. (2006), we can obtain the score vector S. We now compute $\text{cov}(S)$. It is easy to show that

$$\text{var}(S_1) = \text{var}\left[\sum_{i=1}^{n} \frac{1}{\sigma^2}(Y_i - \bar{Y})C_i\right] = \frac{1}{\sigma^2}\sum_{i=1}^{n} C_i^2.$$

For the second component, we use matrix notation. Let $A = (X - \bar{X})(X - \bar{X})^T$, and let $V = \text{cov}(Y)$. Under the null, we have $V = \sigma^2 I_n$. From

$$\begin{aligned} S_2 &= \frac{1}{2\sigma^4}U^T U - \frac{1}{2\sigma^2}\text{trace}(I_U) \\ &= \frac{1}{2\sigma^4}(Y - \bar{Y})^T A (Y - \bar{Y}) - \frac{1}{2\sigma^2}\text{trace}(A), \end{aligned}$$

we obtain

$$\begin{aligned} \text{var}(S_2) &= \frac{1}{4\sigma^8}\text{var}[(Y - \bar{Y})^T A (Y - \bar{Y})] \\ &= \frac{1}{4\sigma^8}2\text{trace}(AVAV) \\ &= \frac{1}{2\sigma^4}\text{trace}(A^2). \end{aligned}$$

To compute the covariance, note that

$$\begin{aligned} \text{cov}(S_1, S_2) &= \text{cov}\left[\frac{1}{\sigma^2}(Y - \bar{Y})^T C, \frac{1}{2\sigma^4}(Y - \bar{Y})^T A (Y - \bar{Y})\right] \\ &= \frac{1}{2\sigma^6}C^T E[(Y - \bar{Y})(Y - \bar{Y})^T A (Y - \bar{Y})] = 0. \end{aligned}$$

The last equality follows by examining each element in the expectation. For example, the first element is

$$
\begin{aligned}
& E[(Y_1 - \bar{Y})(Y - \bar{Y})^T A(Y - \bar{Y})] \\
= \ & \text{trace}\{AE[(Y - \bar{Y})(Y - \bar{Y})^T(Y_1 - \bar{Y})]\} + E[(Y_1 - \bar{Y})(Y - \bar{Y})^T]AE(Y - \bar{Y}) \\
= \ & 0.
\end{aligned}
$$

Next we treat the binary outcome. It is easy to see that $\text{var}(S_1) = p(1-p)C^T C$. To compute $\text{var}(S_2)$, note that

$$
\begin{aligned}
\text{var}(U^T U) &= \text{var}\Big\{ \sum_k \Big[\sum_i (Y_i - \bar{Y}) C_{ik} \Big]^2 \Big\} \\
&= \text{var}\Big\{ \sum_k \Big[\sum_i (Y_i - \bar{Y})^2 X_{ik}^2 + \sum_{i \neq j} (Y_i - \bar{Y})(Y_j - \bar{Y}) C_{ik} C_{jk} \Big] \Big\}.
\end{aligned}
$$

Since Y_i is a Bernoulli random variable, it can be shown that under the null

$$
\text{var}\Big[\sum_i (Y_i - \bar{Y})^2 C_{ik}^2 \Big] = p(1-p)[1 - 6p(1-p)] \sum_i C_{ik}^4 - \sum_i [p(1-p)C_{ik}^2]^2,
$$

and

$$
\text{var}\Big[\sum_{i \neq j} (Y_i - \bar{Y})(Y_j - \bar{Y}) C_{ik} C_{jk} \Big] = 4p^2(1-p)^2 \sum_{i=1}^{n} \sum_{j=i+1}^{n} C_{ik}^2 C_{jk}^2.
$$

Lastly, we replace p by $\bar{Y}$ to obtain the variance estimate under the null and the score statistic (5) for the binary data.
